# Association Between Body Mass Index and the Composition of Leucocyte-Poor Platelet-Rich Plasma: Implications for Personalized Approaches in Musculoskeletal Medicine

**DOI:** 10.3390/cimb47100824

**Published:** 2025-10-08

**Authors:** Hadrian Platzer, Alena Bork, Malte Wellbrock, Ghazal Pourbozorg, Simone Gantz, Reza Sorbi, Ravikumar Mayakrishnan, Sébastien Hagmann, Yannic Bangert, Babak Moradi

**Affiliations:** 1 Department of Orthopedics and Trauma Surgery, University Medical Center Schleswig-Holstein, Campus Kiel, Arnold-Heller-Straße 3, 24105 Kiel, Germany; hadrianmarius.platzer@uksh.de (H.P.);; 2 Orthopedic Research Center, Kiel University, Michaelisstr. 5, 24105 Kiel, Germany; 3 Department of Orthopedics, Heidelberg University Hospital, Schlierbacher Landstraße 200a, 69118 Heidelberg, Germanyyannic.bangert@med.uni-heidelberg.de (Y.B.)

**Keywords:** BMI, cytokines, growth factors, platelets, platelet-rich plasma, PRP

## Abstract

Platelet-rich plasma (PRP) has gained attention in regenerative medicine due to its bio-active proteins with tissue-healing potential. However, heterogeneity in PRP composition remains a major challenge for reproducibility and standardization. Given that body mass index (BMI) affects systemic blood parameters, we investigated whether BMI affects the cellular and molecular composition of PRP. Seventy-three participants were stratified into normal weight, overweight, and obese groups. PRP was prepared using a double-syringe system, and platelet activation was induced by freeze–thaw cycles. Whole blood and PRP cell counts were analyzed, and IL6, IGF1, HGF, and PDGF-BB levels in PRP were quantified by ELISA. Platelet enrichment and levels of IGF1, HGF, and PDGF-BB in PRP did not significantly differ between BMI groups. In contrast, IL6 concentrations were higher in normal-weight compared to overweight and obese individuals. Moreover, BMI-related associations emerged between platelet counts and PDGF-BB, and between PRP proteins and sex or caffeine intake, suggesting a more complex BMI-specific modulation of PRP composition. In conclusion, our findings support considering BMI as a relevant factor in PRP therapy. Incorporating BMI into PRP standardization strategies could improve reproducibility and support personalized regenerative approaches.

## 1. Introduction

Platelet-rich plasma (PRP) is an autologous, blood-derived concentrate with supraphysiological platelet levels that release a diverse range of cytokines and growth factors upon activation [1]. These bioactive mediators are believed to stimulate anabolism and reduce inflammatory catabolism, making PRP a promising regenerative therapy in sports and musculoskeletal medicine. It is widely used to treat tendon and muscle injuries, cartilage defects, and osteoarthritis (OA) [2], the most common joint disease worldwide [3]. The lack of disease-modifying treatments for OA has further fueled interest in PRP as a regenerative therapy. Despite its clinical popularity, the clinical effectiveness of PRP for sports-related injuries and osteoarthritis remains inconclusive due to inconsistent findings across preclinical and clinical studies [4,5,6,7,8]. An umbrella review highlighted substantial heterogeneity in study results, stressing the urgent need for standardized protocols to clarify PRP’s therapeutic role [9]. Major clinical guidelines such as those from the American College of Rheumatology (ACR) and the Osteoarthritis Research Society International (OARSI) currently do not recommend PRP for hip or knee OA [10] due insufficient robust evidence. The inconsistent clinical outcomes of PRP therapy arise from multiple factors, including poorly understood mechanisms of action involving its complex mixture of bioactive proteins [4,5,6,8,11] and methodological variability such as differing preparation protocols [12]. In addition, patient-specific characteristics likely play a critical role in influencing PRP composition and function but remain largely unexplored to date [13,14]. One such potential factor is body mass index (BMI), known to affect hematological parameters, systemic inflammatory status and protein levels [15], all of which could impact the regenerative capacity of PRP. Previous studies investigating the role of BMI in PRP have yielded conflicting results: some report no significant influence on PRP characteristics or clinical efficacy [16,17,18], while others suggest better sustained benefits in individuals with lower BMI [19,20].

A key limitation of these studies is the lack of standardized BMI stratification and the insufficient consideration of demographic and lifestyle factors that may modulate PRP composition in the context of BMI.

This prospective study addresses these gaps by investigating the impact of BMI on the cellular and protein composition of leucocyte-poor platelet-rich plasma (LP-PRP), with a particular focus on platelet concentrations and key bioactive mediators involved in tissue repair, inflammation, and metabolic regulation. To this end, we analyzed levels of interleukin-6 (IL6), hepatocyte growth factor (HGF), platelet-derived growth factor-BB (PDGF-BB), and insulin-like growth factor-1 (IGF1). IL6 is a key immunomodulatory cytokine mainly involved in regenerative processes including tendon repair, OA pathophysiology and is elevated in obesity-associated inflammation [15,21,22]. HGF, another important tissue repair mediator, is frequently dysregulated in metabolic disorders [23,24]. PDGF-BB promotes cell proliferation and matrix remodeling [25,26] and IGF1, known for its anabolic potential, exhibits BMI-dependent systemic variation [27,28].

Beyond quantifying LP-PRP platelet concentrations and protein levels across BMI groups, we examined correlations with lifestyle and demographic factors—including sex, age, alcohol use, and caffeine consumption—as potential contributors to BMI-associated variability. By exploring these relationships in a well-characterized cohort, we seek to provide new insights and possibly support the development of more standardized PRP protocols and improve their clinical application and outcomes in regenerative sports and musculoskeletal medicine.

## 2. Materials and Methods

### 2.1. Study Cohort

This observational, descriptive study included 73 participants (33 males, 40 females), with a mean age of 28.70 ± 4.53 years. For comparative purposes, participants were classified into three BMI categories according to the World Health Organization (WHO) criteria with normal weight (BMI 18.5–24.9), overweight (BMI 25.0–29.9) and obesity (BMI ≥ 30) to ensure comparability across studies. Underweight participants (BMI < 18.5 kg/m^2^) were not included, as this subgroup is often affected by malnutrition or chronic disease, including associated medication use, which could act as potential confounders. Extensive exclusion criteria were defined, and no study participant met any of these criteria. Exclusion criteria comprised (1) history of malignancy, chemotherapy, or hematologic disorders; (2) acute or chronic immune disease; (3) surgery within the past six months; (4) acute musculoskeletal injury; (5) intake of disease-modifying antirheumatic drugs (DMARDs) or corticosteroids within the last three months; (6) intake of non-steroidal anti-inflammatory drugs (NSAIDs) or paracetamol within the last six weeks; and (7) intake of acetylsalicylic acid (ASA) within the last two weeks.

No participant included in this study showed clinical signs of acute or chronic inflammation, as assessed by physical examination and whole blood analysis. Three individuals with laboratory findings indicative of infection were therefore excluded from further analysis and not included in the study population (Table 1).

Between BMI groups, alcohol consumption differed significantly (χ^2^ test, *p* = 0.031), but there were no differences regarding age, sex and caffeine intake. All participants provided written informed consent prior to participation. The study protocol was approved by the Ethics Committee of the University of Heidelberg (S-631/2021) on 15 September 2021.

### 2.2. Blood Sampling

Venous blood was collected from a peripheral arm vein, with 2.7 mL drawn into EDTA tubes (S-Monovette^®^ EDTA K3E; Sarstedt, Nümbrecht, Germany) for subsequent hematological analysis. In parallel, 15 mL of whole blood were collected for PRP preparation using a double-syringe system (Arthrex ACP System; Arthrex, Naples, FL, USA). Immediate processing was performed to standardize the sample handling and reduce methodological errors.

### 2.3. LP-PRP Preparation, Hematological Profiling

LP-PRP was prepared according to the manufacturer’s guidelines [29]. Blood was drawn via the ACP anticoagulant free double syringe system and centrifuged at 1500 rpm for 5 min (Horizon 24-AH, Drucker Diagnostics, Port Matilda, PA, USA), yielding approximately 5–6 mL of LP-PRP.

An automated hematology analyzer from Sysmex XN-1000 (Sysmex Corporation, Kobe, Japan) quantified platelets, erythrocytes, and leukocytes in whole blood and LP-PRP. This confirmed normal whole blood parameters without infectious or inflammatory signs and verified platelet enrichment in LP-PRP.

The resulting LP-PRP was aliquoted into 1.5 mL Eppendorf tubes (Eppendorf AG, Hamburg, Germany) and stored at −80 °C (Dometic UF 755 GG Ultra Low Temperature Freezer; Dometic, Solna, Sweden) within 30 min post-preparation for later protein analysis. To induce the release of growth factors and other bioactive molecules from platelets contained in PRP, samples were subjected to two freeze–thaw cycles to induce platelet protein release as described before [30]. Prior to the first thaw, PRP was treated with 20 µL of unfractionated heparin-natrium (25,000 I.U./5 mL; LEO Pharma A/S, Ballerup, Denmark) per 1 mL of sample to prevent coagulation.

Before protein analysis, PRP samples were thawed at room temperature and centrifuged using a filtration plate (AcroPrep Advance 96-Well Plate, 350 µL, 3 µm glass fiber/0.2 µm Supor Short Tip Natural PP Base Membrane; Pall Corporation, Port Washington, NY, USA) at 1400× *g* for 10 min to remove cellular debris from lysed platelets. All PRP samples were handled identically to ensure consistency.

### 2.4. Protein Quantification

The concentrations of selected cytokines and growth factors—IL6, IGF1, HGF, and PDGF-BB—were measured by enzyme-linked immunosorbent assay (ELISA) following the manufacturers’ protocols (R&D Systems, Minneapolis, MN, USA, lot number IGF1: P310291; lot number IL6: P320482 and Sigma Aldrich, St. Louis, MI, USA; lot number PDGF-BB-0307J180; HGF-0308K0201). The absorbance was immediately read at 450 nm using an Autobio Labtec Instruments Co, Microplate photometer (Software: AUTOsoft, version 2.6.9; Zhengzhou, China).

### 2.5. Statistical Analysis

Continuous variables are summarized as mean (±standard deviation) with interquartile range (IQR), while categorical variables are reported as counts (percentages), as specified. To examine the independence between two categorical variables in a contingency table, the Chi-Square Test was performed. The normality of continuous data was detected using the Shapiro–Wilk test.

Given the non-normal distribution of the data, the non-parametric Kruskal–Wallis ANOVA (or Kruskal–Wallis H test) was performed to assess whether there were statistically significant differences in blood cell counts and protein components across the BMI groups. When necessary, post hoc Kruskal–Wallis tests with Bonferroni corrections were applied to account for multiple comparisons, thereby minimizing the risk of Type I errors. For correlation analyses, Spearman’s rank correlation coefficient (ρ) was used when one or both variables deviated from a normal distribution or when a non-linear relationship was assumed. Dichotomous variables and metric variables were analyzed using the point-biserial correlation (r). All tests were two-tailed, and statistical significance was defined as a *p*-value < 0.05. Statistical analyses were performed using SPSS version 29.0.2.0 (IBM Corp., Armonk, NY, USA). Graphs were constructed using GraphPad Prism, version 10 (GraphPad Software, La Jolla, CA, USA).

## 3. Results

### 3.1. BMI and Its Association with Whole Blood and LP-PRP Cellular Profiles

BMI was significantly associated with variations in whole blood cell populations. Leukocyte counts increased progressively from normal weight to obese individuals (*p* < 0.001), while erythrocyte counts were significantly higher in overweight individuals compared to those of normal weight (*p* = 0.002). The concentrations of the individual subgroups of leukocytes, including lymphocytes, monocytes, basophils, and eosinophils, did not differ significantly between BMI categories (Table 2).

LP-PRP samples were low in erythrocytes (mean ± SD: 0.03 ± 0.04) and leukocytes (mean ± SD: 0.06 ± 0.04). Comparative analysis of leukocyte levels in PRP between BMI groups revealed no statistical differences. In addition, no significant correlation between leukocyte levels in PRP and IL6 were determined.

**Figure 1:**
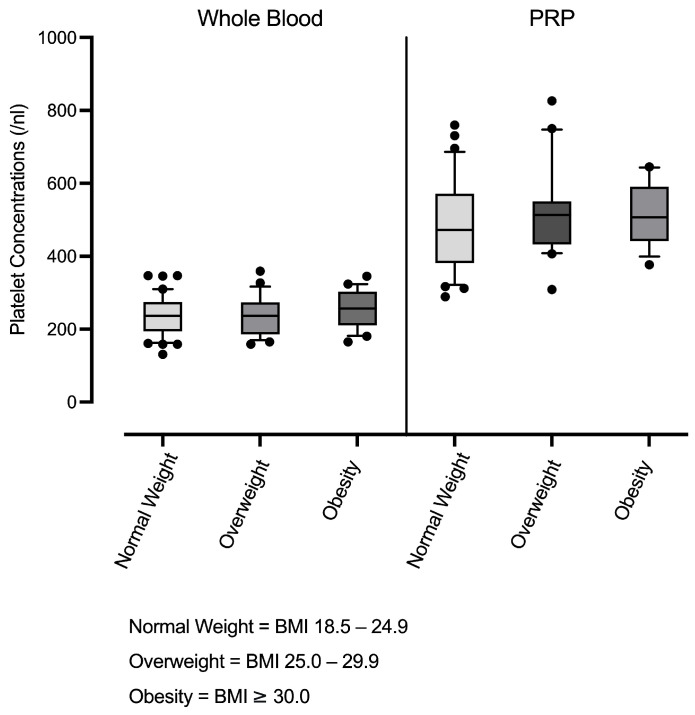
Platelet concentrations in whole blood and leucocyte-poor PRP (LP-PRP) across BMI groups (normal weight n = 37; overweight n = 20; obesity n = 16). Box-and-whisker plots are shown (units:/nL).

Furthermore, hematological analysis across BMI groups revealed no significant differences either in platelet counts in whole blood or LP-PRP (Figure 1), or in the PRP-to-whole blood platelet ratio, indicating that the degree of platelet enrichment in PRP remained stable across BMI categories. The mean PRP-to-whole blood platelet ratio was 2.08 ± 0.35 (min: 1.59, max: 3.05, Table 2).

### 3.2. Growth Factor and Cytokine Levels in LP-PRP Stratified by BMI

**Figure 2:**
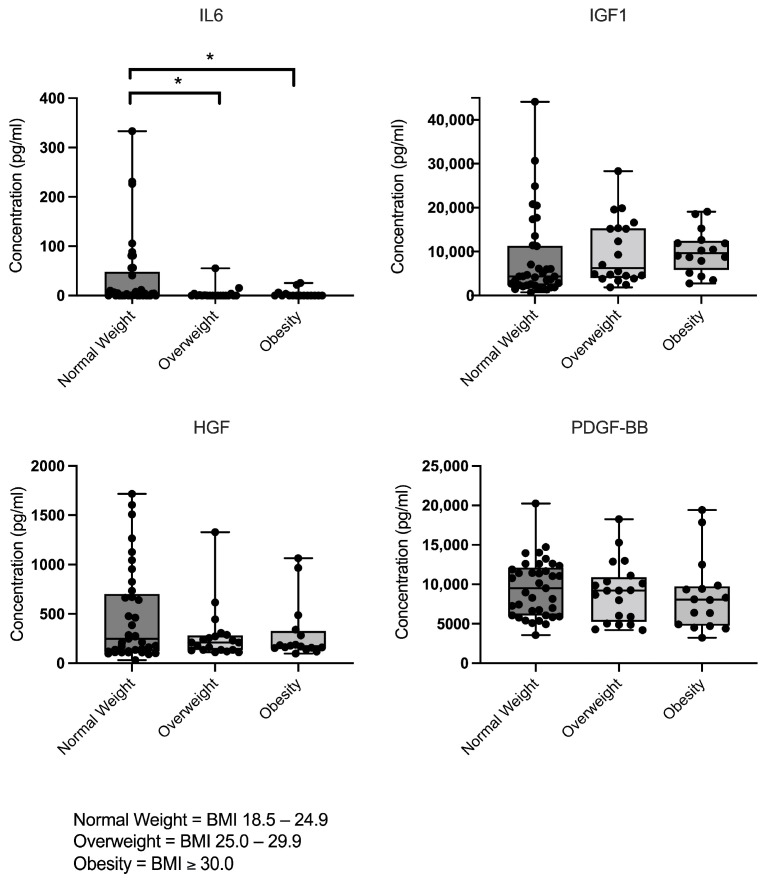
Concentrations of IL6, IGF1, HGF, and PDGF-BB in leucocyte-poor PRP (LP-PRP) stratified by BMI group (normal weight *n* = 37; overweight *n* = 20; obesity *n* = 16), as measured by ELISA. The data are presented as individual data points overlaid on box-and-whisker plots (showing minimum to maximum values) (units: pg/mL). Significant differences are indicated by asterisks (*p* < 0.05).

Overall, interindividual variability in cytokine and growth factor concentrations were detected. Despite the inclusion of a broad range of clinical and demographic parameters and the application of strict exclusion criteria, no parameters in the raw dataset explained the observed protein-associated data variability. Mean levels of growth factors IGF1, HGF, and PDGF-BB remained consistent across individuals with normal weight, overweight and obesity. In contrast, IL6 levels showed significant differences between BMI groups. Individuals with normal BMI exhibited significant higher IL6 levels compared to those who were overweight or obese (Figure 2).

### 3.3. BMI-Related Variations in Correlations Between Platelets and Proteins

Further subgroup analyses explored associations between platelet concentrations in whole blood and LP-PRP with IL6, IGF1, HGF, and PDGF-BB across the different BMI categories. As shown in Table 3, Spearman correlation analyses revealed variable but mostly non-significant associations between platelet counts and protein concentrations across BMI subgroups.

Notably, a moderate positive correlation reached significance in the normal-weight group between platelet counts and PDGF-BB levels in LP-PRP. For the other proteins, correlation directions varied across BMI groups, although these associations did not reach statistical significance. Interestingly, PDGF-BB was the only protein showing consistent positive correlations independent of BMI.

### 3.4. BMI-Related Variations in Correlations Between Lifestyle or Demographic Parameters and PRP Proteins

No significant correlations were found between age or alcohol use and any of the analyzed proteins, including in BMI-stratified analyses. BMI-specific correlations for sex and caffeine consumption are summarized in Table 4. Among normal-weight individuals HGF levels were significantly negative correlated with sex, while HGF showed a significant positive correlation with caffeine intake. In the overweight group, a significant association was detected between IGF1 levels and sex. In contrast, no significant correlations were observed between sex or caffeine and protein levels in the obese cohort (Table 4). Beyond these findings, weak correlations between sex or caffeine intake and PRP protein concentrations were observed but did not reach statistical significance. Notably, with the exception of PDGF-BB, which showed a BMI-independent negative correlation with sex, the direction of other correlations appeared to vary systematically across BMI groups.

## 4. Discussion

This study examined the relationship between body mass index (BMI) and the composition of LP-PRP, focusing on platelet enrichment and key proteins relevant to tissue regeneration and inflammation. Demographic and lifestyle factors—often overlooked in PRP research—were systematically analyzed to assess BMI-associated interactions. While BMI was not directly associated with platelet counts or growth factor concentrations, it significantly affected IL6 levels and altered correlations between PRP proteins and platelet counts, demographic and lifestyle variables. These findings suggest that BMI modulates LP-PRP composition, which may in turn influence its therapeutic efficacy. Nevertheless, the present work is descriptive in nature and should be interpreted as hypothesis-generating, without drawing direct conclusions on clinical outcomes.

By addressing a critical gap in the literature, this study highlights the relevance of metabolic, demographic and lifestyle factors in PRP biology—particularly relevant to sports and musculoskeletal medicine. Despite widespread orthopedic use, PRP clinical outcomes remain heterogeneous, likely due to variability in formulations and insufficient individual-level consideration. Analyzing a well-characterized, medication-free cohort, this work supports more standardized and personalized treatment approaches.

In this study, BMI showed no significant association with platelet counts in whole blood, which is consistent with previous findings [31,32]. However, the existing literature remains mixed [15,33], likely due to differences in study populations with regard to demographics and medical histories. Similarly, BMI did not significantly affect platelet counts in LP-PRP aligning with earlier reports [18,32]. This is encouraging, as platelets represent the primary source of the cytokines and growth factors believed to mediate PRP’s therapeutic effects—particularly in LP-PRP. However, protein concentrations in LP-PRP are not solely determined by platelet counts but are also likely influenced by platelet activation status and functional capacity [34]. To account for this, we additionally quantified key proteins involved in tissue regeneration and inflammation that are relevant to musculoskeletal conditions.

In line with the stable platelet counts observed across BMI groups, no significant differences were found in PRP concentrations of IGF1, HGF, and PDGF-BB. These findings suggest that the core regenerative potential of LP-PRP—primarily mediated by platelet-derived growth factors—is likely preserved across different body compositions, when potential BMI-related interactions with demographic or lifestyle factors are not considered (as discussed below). Thus, our results are consistent with previous observations reporting no significant differences in PRP growth factor levels between obese and non-obese patients [34].

Despite stable platelet counts and comparable growth factor concentrations across BMI groups, this study identified significant differences in IL6 concentrations in LP-PRP. IL6, a central immunomodulatory cytokine [34], was unexpectedly elevated in normal-weight individuals compared to those with obesity. As IL6 plays a key role in immune regulation and tissue repair, this finding may be clinically relevant for PRP-based musculoskeletal therapies. For example, IL6 is critical for tendon repair [34], indicating that its content in PRP could significantly impact efficacy in treating of tendon injuries. Notably, IL6 in PRP is not derived from platelets but primarily originates from plasma and leukocytes [34]. The inverse association observed in this study contrasts with the well-documented elevation of systemic IL6 levels in obesity, driven by chronic low-grade adipose tissue inflammation [34]. This discrepancy suggests that the cytokine profile of LP-PRP may diverge fundamentally from that of peripheral blood. Our findings indicate that even in leukocyte-poor preparations, residual leukocytes may shape the cytokine milieu, and that BMI may influence IL6 via functional rather than numerical immune cell alterations. Obesity is known to impair leukocyte functionality; for example, monocytes from pregravid obese individuals release less IL6 upon LPS stimulation than those from normal-weight subjects [34]. While our study was not designed to assess leukocyte function directly, these results highlight the need for further mechanistic investigations. Future studies should explore whether the BMI-IL6 association is more pronounced in leukocyte-rich PRP, particularly since findings of whole blood analysis in this study showed increased leukocyte counts in higher BMI individuals, which may alter LR-PRP composition.

In this study, only one significant association was detected between platelets and PRP proteins: a positive correlation between platelet counts in PRP and PDGF-BB concentrations in the normal-weight group. This finding suggests that obesity-related metabolic alterations may impair platelet function or disrupt PDGF-BB release in PRP, underscoring the BMI-dependent modulation of PRP composition. PDGF-BB is predominantly released from platelet α-granules [34], yet residual leukocytes may also indirectly modulate its bioavailability through paracrine signaling or interactions with platelet activation. In addition, several non-significant correlations between platelet counts (in whole blood and PRP) and PRP protein concentrations were observed, with their direction varying across BMI groups. Our findings are consistent with previous reports showing that correlations between platelet counts and growth factor levels are inconsistent [35], supporting the notion that platelet count alone is insufficient as a quality control parameter for PRP and that multi-parameter approaches should be considered. Together, these findings underscore the need for mechanistic studies to elucidate how obesity might interfere with cellular contributors to protein release in PRP.

Additionally, correlations of demographic and lifestyle factors with PRP composition were associated with BMI categories. While age and sex were balanced across groups, BMI-specific significant associations emerged: sex correlated significantly with growth factor levels only in normal weight and overweight individuals. This adds a new layer to prior findings on sex differences in PRP composition [34], indicating a moderating role of BMI. Similarly, caffeine intake was associated significantly with changes in PRP composition, but only in normal-weight individuals.

Together, these findings highlight the complex interplay between BMI, demographic and lifestyle factors, as their interplay may shape the profile of bioactive molecules in LR-PRP.

This study was intentionally designed as an exploratory, descriptive analysis in a healthy, medication-free cohort to establish a baseline of PRP composition with minimal confounding from disease or treatment. Assessing clinical outcomes was therefore not appropriate at this stage; rather, these data provide a hypothesis-generating framework for subsequent patient-based studies. In this regard, the present work should be viewed as the initial step of a planned research sequence. By systematically characterizing BMI-associated variability in PRP composition, we provide the groundwork for subsequent mechanistic and clinical validation studies. Such investigations will be necessary to establish causal relationships, assess clinical efficacy, and ultimately ensure the translational relevance of personalized PRP protocols. Although we did not evaluate clinical endpoints, by analyzing key proteins relevant to musculoskeletal pathology and recovery, our findings suggest that BMI-associated variability in PRP composition may reflect differences in its intrinsic quality. Because PRP molecular composition is closely linked to biological activity, such alterations are likely to influence therapeutic potential. For example, the altered levels of IL6 observed in this study—a cytokine with central immunomodulatory functions that can exert either regenerative or pathological effects depending on disease context and the induced signaling pathway—suggest that BMI-associated variability also affects the therapeutic potential of PRP [21,22].

Taken together, these observations emphasize that LP-PRP is unlikely to be a one-size-fits-all therapy. Individual characteristics—such as BMI, sex, and caffeine intake—significantly shape its molecular composition, providing a rationale for future clinical studies that integrate PRP profiles with treatment outcomes and for the development of personalized PRP-based interventions. Beyond characterizing this variability, it will be important to elucidate the mechanisms governing PRP activity to guide optimization strategies. Building on such mechanistic insights, several approaches are being explored to enhance PRP activity, including modifications of preparation protocols, supplementation with bioactive factors, and the use of alternative PRP sources [36,37]. These concepts highlight translational pathways to improve the therapeutic efficacy of PRP in diverse patient populations.

This study has several strengths. It provides a detailed evaluation of BMI-related effects on PRP composition in a well-characterized cohort of individuals, thereby minimizing the confounding influence of comorbidities or medication use. Moreover, by simultaneously assessing demographic and lifestyle factors, this work offers a more differentiated understanding of individual-level factors that may modulate PRP composition.

However, several limitations of this study should be acknowledged. Our descriptive design in a healthy cohort was chosen to minimize confounding and to establish baseline variability in PRP composition; evaluating clinical outcomes was beyond the scope and not meaningful in this population. Future patient-based studies are required to test whether the observed compositional differences and associations translate into differential clinical efficacy. Although the sample size was adequate for exploratory analyses, it limits the generalizability of the findings and precludes definitive clinical conclusions. In particular, non-significant trends such as the observed variation in HGF concentrations across BMI groups might require larger cohorts to be clarified. Further, this study focused exclusively on leucocyte-poor PRP, and the findings may not be transferable to other PRP formulations such as leucocyte-rich PRP, which differs in cellular composition and cytokine profile. While we assessed key proteins relevant to tissue regeneration and inflammation, other biologically active components in PRP potentially modulated by BMI were not evaluated and warrant a further extensive investigation. Lastly, BMI does not differentiate between fat and lean mass, nor does it capture fat distribution. More refined measures such as body fat percentage, may provide a more accurate assessment of individual metabolic status and should be considered in future studies.

## 5. Conclusions

This study supports the rationale for personalizing PRP therapies based on individual characteristics—particularly in sports and musculoskeletal medicine, where treatment efficacy may be modulated by BMI. Although BMI did not affect platelet enrichment or concentrations of key growth factors in LP-PRP, it was associated with significant variation in IL6 levels, indicating an impact on the cytokine profile. In addition, BMI-specific correlations emerged between LP-PRP protein concentrations and variables such as platelet count, sex, and caffeine intake. These results highlight the biological complexity of PRP and reinforce that LP-PRP is not a compositionally uniform product. Rather, the interplay of patient-specific factors and habits jointly shapes its molecular landscape. While exploratory in nature, these findings provide a biological rationale for future mechanistic and clinical studies aimed at clarifying the translational relevance of BMI-associated variability in PRP composition, and they may also offer a plausible biological explanation for the heterogeneous treatment responses observed between lean and obese individuals.

## Figures and Tables

**Table 1:** Demographic and lifestyle characteristics of the study population stratified by BMI groups.

	Total ^1^ (*n* = 73)	BMI Groups			*p*-Value
		Normal Weight (*n* = 37)	Overweight (*n* = 20)	Obesity (*n* = 16)	
Sex, *n* (%)					0.053
Female	40 (55%)	21	7	12	
Male	33 (45%)	16	13	4	
Age (years), mean ± SD; IQR	28.70 ± 4.53; 8.00	27.62 ± 4.09; 6.00	28.85 ± 4.06; 8.00	31.00 ± 5.37; 10.00	0.082
BMI (kg/m^2^), mean ± SD; IQR	25.85 ± 6.90; 8.68	20.80 ± 1.73; 3.11	26.60 ± 1.30; 1.78	36.60 ± 5.68; 9.57	–
Lifestyle factors, *n* (%)					
Alcohol use	39 (53%)	19	15	5	0.031
Caffeine use	42 (58%)	20	14	8	0.410

^1^ Continuous variables are presented as mean ± standard deviation (SD) and interquartile range (IQR); categorical variables are shown as absolute and relative frequencies; *p*-Values were calculated using the Chi-square test for categorical variables and Kruskal–Wallis ANOVA for continuous variables. Overall significance levels are shown in the rightmost column.

**Table 2:** Cell analysis in whole blood and LP-PRP stratified by BMI groups.

			BMI Groups ^1^						*p*-Value
			Normal Weight		Overweight		Obesity		
			Mean	SD	Mean	SD	Mean	SD	
Whole blood	Platelets (/nL)		237.32	53.57	243.30	55.23	264.44	50.14	0.264
	Erythrocytes (×10^3^/nL)		4.59	0.44	4.95	0.24	4.77	0.47	0.002
	Leukocytes (/nL)		5.61	1.35	6.18	1.15	8.14	2.34	<0.001
	Lymphocytes (%)		32.78	6.38	31.09	7.84	28.27	5.51	0.082
	Monocytes (%)		5.51	1.39	5.82	1.62	5.33	1.70	0.725
	Basophils (%)		0.65	0.28	0.66	0.22	0.68	0.24	0.804
	Eosinophils (%)		2.85	2.52	2.71	1.80	2.33	1.15	0.864
	Neutrophils (%)		55.87	6.70	57.63	9.24	61.43	6.74	0.057
PRP	Platelets (/nL)		487.89	126.51	527.60	130.93	515.50	84.93	0.450
PRP-to-whole blood platelet ratio			2.07	0.35	2.10	0.38	1.97	0.27	0.227

^1^ Mean values and standard deviations (SD) of cell concentrations and corresponding *p*-values from comparative analyses between BMI groups are shown. Leukocyte subpopulations are presented as percentages (%) of the total leukocyte population.

**Table 3:** Correlation between LP-PRP protein and platelet concentrations in Whole Blood and LP-PRP across BMI groups.

		Platelets Whole Blood ^1^			Platelets PRP ^1^		
		Normal Weight	Overweight	Obesity	Normal Weight	Overweight	Obesity
IL6	ρ *p*-Value	−0.176	0.235	0.168	0.970	0.207	−0.330
		0.298	0.318	0.534	0.570	0.382	0.904
IGF1	ρ *p*-Value	0.148	0.411	−0.082	0.278	0.155	−0.165
		0.382	0.072	0.762	0.095	0.515	0.542
HGF	ρ *p*-Value	−0.274	0.090	−0.326	0.087	0.179	−0.200
		0.101	0.707	0.217	0.607	0.449	0.458
PDGF-BB	ρ *p*-Value	0.165	0.375	0.274	0.364 *	0.380	0.247
		0.330	0.103	0.305	0.027	0.098	0.356

^1^ Correlations are shown for mean IL6, IGF1, HGF, and PDGF-BB concentrations (pg/mL) in LP-PRP with platelet concentrations (/nL) in whole blood and LP-PRP. Spearman’s rank correlation coefficients (ρ) and corresponding *p*-values are reported for each BMI category. Significant correlations are indicated by asterisks: * *p* < 0.05.

**Table 4:** Correlation between sex or caffeine intake and LP-PRP protein concentrations across BMI groups.

	IL6	IGF1	HGF	PDGF-BB
	Normal Weight			
sex r	−0.140	−0.171	−0.324 *	−0.306
*p*-Value	0.407	0.312	0.050	0.066
caffeine r	0.268	0.308	0.459 **	−0.099
*p*-Value	0.109	0.064	0.004	0.561
	Overweight			
sex r	0.274	0.667 **	0.190	−0.065
*p*-Value	0.242	0.001	0.422	0.787
caffeine r	−0.174	−0.282	−0.208	0.421
*p*-Value	0.463	0.229	0.378	0.065
	Obesity			
sex r	0.261	0.356	0.199	−0.043
*p*-Value	0.328	0.176	0.459	0.875
caffeine r	−0.359	−0.054	−0.393	0.045
*p*-Value	0.172	0.842	0.132	0.870

^1^ Data are presented as point-biserial correlation coefficients (r) with corresponding *p*-values. Significant correlations are indicated by asterisks: * *p* < 0.05, ** *p* < 0.01.

## Data Availability

The data presented in this study are available from the corresponding author upon reasonable request and subject to institutional and ethical approval.

## Abbreviations

The following abbreviations are used in this manuscript:

ACR	American College of Rheumatology
ANOVA	Analysis of Variance
ASA	Acetylsalicylic Acid
BMI	Body Mass Index
DMARD	Disease-Modifying Anti-Rheumatic Drug
DMOAD	Disease-Modifying Osteoarthritis Drug
EDTA	Ethylenediaminetetraacetic Acid
ELISA	Enzyme-Linked Immunosorbent Assay
HGF	Hepatocyte Growth Factor
IGF	Insulin-like Growth Factor
IL	Interleukin
IQR	Interquartile Range
LP-PRP	Leukocyte-Poor Platelet-Rich Plasma
LR-PRP	Leukocyte-Rich Platelet-Rich Plasma
mL	milliliter
MMP	Matrix Metalloproteinase
nL	nanoliter
NSAID	Non-Steroidal Anti-Inflammatory Drug
OA	Osteoarthritis
OARSI	Osteoarthritis Research Society International
PDGF	Platelet-Derived Growth Factor
pg	picogram
PRP	Platelet-Rich Plasma
SD	Standard Deviation
SPSS	Statistical Package for the Social Sciences
WHO	World Health Organization
